# Retroviruses 2004: Review of the 2004 Cold Spring Harbor Retroviruses conference

**DOI:** 10.1186/1742-4690-1-25

**Published:** 2004-09-09

**Authors:** Eric O Freed, Susan R Ross

**Affiliations:** 1 Virus-Cell Interaction Section, HIV Drug Resistance Program, National Cancer Institute at Frederick, National Institutes of Health, Bg. 535/Rm. 108, Frederick, MD 21702-1201, USA; 2 University of Pennsylvania School of Medicine, Room 313 BRBII/III, 421 Curie Blvd., Philadelphia, PA 19104-6142, USA

## Abstract

For the past several decades, retrovirologists from around the world have gathered in late May at the Cold Spring Harbor Laboratories in New York to present their studies in formal talks and posters, and to discuss their ongoing research informally at the bar or on the beach. As organizers of the 2004 Cold Spring Harbor Retroviruses Conference, we have been asked by the editors of Retrovirology to prepare a review of the meeting for publication on-line. Our goal in this review is not to provide a detailed description of data presented at the meeting but rather to highlight some of the significant developments reported this year. The review is structured in a manner that parallels the organization of the meeting; beginning with the entry phase of the replication cycle, proceeding with post-entry events, assembly and release, integration, reverse transcription, pathogenesis/host factors, RNA-related events (transcription, processing, export, and packaging) and finishing with antivirals. While the most striking developments this year involved post-entry events and assembly/release, significant progress was made towards elucidating a number of aspects of the retroviral replication cycle.

## Entry

Although no "new" retrovirus receptors were reported at the meeting, several talks centered on recently discovered receptors. N. Manel, from the groups that identified GLUT-1 as an entry receptor for HTLV-1 (N. Taylor, J.-L. Battini, and M. Sitbon) [1], proposed that part of the pathogenic effects of this virus may be due to its perturbation of glucose metabolism. HTLV-1-infected tissue culture cells display decreased glucose uptake as a result of envelope (Env) glycoprotein-GLUT-1 interaction. The authors speculated that if this disruption of glucose metabolism also occurs *in vivo*, it might provide insights into the neuronal damage that occurs in some HTLV-1-infected patients. They also suggested that Env-mediated impairment of GLUT-1 function might contribute to the emergence of preleukemic T cells with new selective advantages [2].

The co-receptor for feline immunodeficiency virus (FIV), perhaps the best non-primate model for HIV-1, has also recently been identified. Work presented by J. Elder showed that FIV preferentially infects certain subsets of T cells through interaction with CXCR4 and a 43 kDa protein. This 43 kDa protein turns out to be CD134, recently demonstrated to be a receptor for FIV [3]. CD134 was first described as a member of the tumor necrosis/nerve growth factor receptor family expressed on activated T cells. By analogy with the role of CD4 in HIV infection, CD134 may target FIV infection to a particular subset of T cells. Extending the CD4/HIV parallel, CD134 may be the molecule that initially engages FIV, followed by CXCR4 binding and virus/cell fusion. Elder suggested that CD134 should be referred to as the FIV attachment receptor and CXCR4 as the entry receptor.

The use of alternative chemokine co-receptors by primary HIV-1 isolates was discussed by S. Neil from R. Weiss's group. They proposed that some primary dual-tropic HIV-1 isolates, especially those isolated from early seroconverters, might infect primary astrocytes, endothelial cells and macrophages through the use of D6, a promiscuous chemokine receptor highly expressed on these cell types. They hypothesized that D6 usage to infect endothelial cells could influence colonization of endothelial compartments and promote placental transmission.

Two groups discussed the risk of pig endogenous retrovirus (PERV) infection of human cells as a potential problem for xenotransplantation. I. Harrison from the Stoye lab showed that recombination between different endogenous PERVs (A and C), which normally have very low titers on human cells, could lead to the production of virus with the capacity to efficiently infect human cells. This tropism change mapped in large part to the Env glycoprotein, but the Gag-Pol region was also implicated. D. Lavillette from the Kabat lab presented evidence that wild-type PERVs, or mutants lacking fully infectious envelopes due to alterations in a conserved PHQ motif (in SU) required for γ-retrovirus infection, could bind and enter human cells when added together with the envelope from gibbon-ape leukemia virus, which does infect human cells. The ability of functional Env glycoproteins from infectious viruses to trans-complement infection by viruses with mutant envelopes or with restricted tropism on particular target cells has been reported for several retroviruses [4,5]. In the case of PERVs, such complementation has the potential of overcoming host-range and interference barriers and could pose a hazard for xenotransplantation.

For a number of years, investigators have attempted to engineer peptide ligands for cell surface receptors into retroviral Env glycoproteins with the goal of designing retroviral gene therapy vectors that would efficiently target specific cell types. These attempts have been hampered by low transduction efficiencies. L. Albritton presented work from her lab demonstrating that incorporation of a peptide sequence into the SU receptor binding site (RBS) may overcome this problem. Her lab constructed a chimera in which the RBS of the Moloney murine leukemia virus (MLV) Env was replaced with the peptide ligand somatostatin (Sst). Such chimeras efficiently infected human cells expressing the Sst receptor, but could no longer infect mouse cells expressing the natural receptor, ATCR1. The similarity in length of the peptide with the sequence it replaced, as well as the structural similarity between ATCR1 and the Sst receptor, may have contributed to the success of this approach, since substitution of the RBS with human stromal-derived factor-1 alpha (SDF-1α), which also uses a structurally similar receptor (CXCR4), has also been successful [6]. In contrast, attempts to make peptide ligand substitutions in this RBS that are either dissimilar in length or use structurally unrelated receptors have met with limited success (i.e. see [7].

Following binding between retroviral Env glycoproteins and their receptors, conformational changes must take place in both the SU and TM that expose the fusion peptide and enable membrane fusion to occur. H. Garoff presented work extending his recently published report [8] showing that for γ-retroviruses this conformational change involves SU-TM disulfide bond isomerization. The isomerization, which is inhibited by Ca^2+^, results in breakage of the SU-TM disulfide bond, the generation of an intra-SU bond, and subsequent exposure of the TM fusion peptide. Garoff speculated that the ability of γ-retroviruses such as MLV to fuse at the cell surface, in contrast to α-retroviruses like ALV that lack isomerization activity and which apparently undergo pH-dependent fusion in a subcellular compartment, is the result of this isomerization.

Several studies have implicated amino acid changes in the cytoplasmic domain of the MLV TM on SU conformation, with consequent effects on viral infectivity and antibody recognition [9,10]. Work presented by R. Montelaro demonstrated that mutations in the cytoplasmic tail of the HIV-1 TM (gp41) can also result in escape from neutralization. Importantly, at least one of these antibody-escape mutants still retained wild-type infectivity, indicating that sequence changes in the TM intracytoplasmic domain should be considered in the characterization of antigenic variants of HIV-1 that escape neutralization.

Dendritic cells express a molecule known as DC-SIGN that binds HIV-1 particles and facilitates their transmission to susceptible target cells [11]. The mechanism by which this molecule promotes the transfer of infectious virions to target cells has been an active area of investigation for the past several years. Work from V. KewalRamani's lab (Wu et al.) showed that DC-SIGN is able to transfer HIV-1 infectivity *in trans *when expressed on some cell types but not when expressed on others. The ability of DC-SIGN to promote virus transfer appears to correlate with the localization of the particles on the DC-SIGN-expressing cell; in cells unable to mediate transfer, virions are internalized, whereas cells able to mediate efficient transfer retain virions at the cell surface. Wu and colleagues also observed that binding and transmission of HIV-1 by immature dendritic cells is Env- and DC-SIGN-dependent, whereas virus binding by mature dendritic cells does not require either Env on the particle or DC-SIGN on the dendritic cell. The molecule(s) expressed on dendritic cells that potentiate DC-SIGN-independent virus transmission await discovery.

## Post-entry events

The last two years have seen major advances in our understanding of the mechanisms by which cells from human and non-human primates restrict retroviral infection. In 2002, Sheehy et al. [12] reported that the HIV-1 accessory protein Vif interacts with the cellular cytidine deaminase APOBEC3G. In the absence of Vif expression, APOBEC3G is incorporated into HIV-1 virions and, during reverse transcription in the target cell, converts cytosines to uracils. This conversion results in the degradation of newly synthesized DNA through the action of host glycosidases and repair enzymes, and leads to G-to-A hypermutation in the newly synthesized viral DNA. Vif counters the antiviral activity of APOBEC3G by blocking its incorporation into virions in the producer cell. Interestingly, Vif displays species specificity in its ability to inactivate APOBEC3G; for example, HIV-1 Vif blocks the antiviral activity of human but not African green monkey APOBEC3G, and SIVagm Vif inactivates African green monkey but not human APOBEC3G. The labs of N. Landau and V. Pathak both reported that the determinant of this species specificity maps to amino acid 128 of APOBEC3G. Data in the Landau lab presentation (Schrofelbauer et al.) suggested that the inability of HIV-1 Vif to block the activity of African green monkey APOBEC3G was due to a lack of binding between these two proteins, whereas the Pathak lab (Xu et al.) reported that mutation of residue 128 of human APOBEC3G did not prevent binding of the mutant protein to Vif. Both labs recently published their findings [13,14].

Since the incorporation of APOBEC3G into virus particles is required for its antiviral activity, several groups have focused on how this cellular protein is incorporated. The mechanism of APOBEC3G incorporation is of particular interest since it appears not to be specific for HIV-1 (e.g., human APOBEC3G is incorporated into MLV particles) and because inhibitors that disrupt Vif's ability to block APOBEC3G incorporation would presumably display antiviral properties. L. Kleiman's lab (Cen et al.) reported that APOBEC3G interacts directly with Gag in a manner dependent on the Gag nucleocapsid (NC) domain. The labs of W. Popik and X.F. Yu also observed that NC plays an important role in the APOBEC3G/Gag interaction. H. Xu from V. Pathak's group reported that mutation of NC reduced but did not abrogate APOBEC3G incorporation into virions. Since NC is a major determinant of RNA packaging into virions, these data, together with the above-mentioned finding that APOBEC3G is incorporated into MLV particles, can be rationalized by a model that proposes an important role for RNA (either viral or cellular) in APOBEC3G incorporation. Such a model would also be consistent with the apparent inability of HIV-1 to evade APOBEC3G incorporation simply by mutation of a specific protein-protein binding site. Interestingly, a study from D. Ho's lab (Simon et al.) found that isolates of HIV-1 that are unable to block APOBEC3G activity are relatively common in infected patients. The authors suggested that sporadic Vif inactivation might be a factor in promoting viral evolution *in vivo *since Vif-defective variants would exhibit a higher mutation rate.

In recent years, several groups have reported that HIV-1 is unable to efficiently infect cells from certain species of Old World monkey (for review see [15]). The block is imposed early post-entry, prior to reverse transcription, and is mediated by an inhibitory factor expressed in Old World monkey cells. The viral determinant of this restriction maps to the capsid (CA) domain of Gag, making this restriction somewhat reminiscent of the Fv1 block described by Lilly and others decades ago [16]. J. Sodroski's group recently published that the rhesus macaque version of the cytoplasmic body component TRIM5α [17] potently blocks HIV-1 infection. Presentations from the labs of J. Sodroski (Stremlau et al.) and P. Bieniasz (Hatziioannou et al.) reported that Ref1 and TRIM5α are species-specific variants of TRIM5α; this finding has now been published [18]. This factor is quite distinct from Fv1, which bears sequence homology to MLV CA [19]. A. Lassaux (from the Battini and Sitbon labs) reported that Fv1 and Ref1 recognize different amino acid combinations within the same 100 amino acid determinant of the MLV CA. Hatziioannou et al. also described their results on characterizing the ability of human- and monkey-derived TRIM5α's to block the entry of a panel of retroviruses. D. Sayah from J. Luban's lab provided a preview of the now-published paper [20] reporting the intriguing finding that in owl monkey cells, the post-entry block to HIV-1 infection is conferred by a TRIM5α-cyclophilin A fusion protein. In another presentation focused on post-entry blocks to retroviral infection, V. KewalRamani's lab (Martin et al.) reported that overexpression of a truncated form of an RNA binding protein that is a component of the poly A machinery blocks an early stage of the HIV-1 infection process without disrupting MLV infectivity.

J. Young (Narayan and Young) reported the development of a cell-free uncoating assay that will likely prove useful in defining the role of cellular factors in stimulating or blocking early post-entry steps in the replication cycle. Avian sarcoma/leukosis virus (ASLV) particles can be trapped in endosomes when cells expressing a GPI-linked version of the ASLV receptor are infected in the presence of NH_4_Cl. The virus-containing endosomes can then be isolated; upon removal of the NH_4_Cl *in vitro*, fusion and reverse transcription take place in a manner dependent upon ATP hydrolysis and the presence of cellular factors. This cell-free uncoating assay can be adapted to other viruses (e.g., HIV-1) by pseudotyping with the ASLV Env glycoprotein. The authors reported that monkey cell restriction factors, and cyclosporin A (which blocks cyclophilin A incorporation into HIV-1 particles), inhibit reverse transcription in this system, suggesting that it faithfully recapitulates certain key aspects of uncoating in infected cells. Some of the data in this presentation were recently published [21].

## Assembly and Release

Major developments have taken place in the past several years in our thinking about the location in the host cell at which retrovirus assembly takes place, and the mechanism by which Gag proteins target the subcellular site of assembly. Until recently, it was felt that viruses that follow the type C assembly pathway [including the lentiviruses (e.g., HIV-1), δ-retroviruses (e.g., HTLV-1), γ-retroviruses (e.g. MLV), etc.] assemble at the plasma membrane. However, it has long been recognized that in some cases (for example, HIV-1 in macrophages) assembly and budding take place at an intracellular compartment. Several groups have recently demonstrated that this compartment is the late endosome or multivesicular body (MVB), and it now appears that while the plasma membrane likely represents the predominant site of assembly for these viruses, Gag can also target and assemble in an endosomal compartment in a variety of cell types. M. Resh's lab (Perlman and Resh) used the tetracyteine/biarsenical live-cell labeling system reported by Gaietta et al. [22] to follow Gag trafficking relatively early (1–4 hrs.) post-synthesis. Their results suggest that Gag may first localize to secretory lysosomes and then subsequently "ride" these vesicles to the plasma membrane. M. Thali's lab recently reported that a significant fraction of HIV-1 Gag colocalizes with late endosomal markers both at intracellular membranes and at the plasma membrane [23]. Together with D. Ott's lab (Nydegger et al.), they have also started using the tetracysteine/biarsenical technology to analyze Gag localization. Like the Resh lab, they observe some association of Gag with late endosomal/MVB membrane, but a significant fraction of newly synthesized Gag appears to associate directly with discrete plasma membrane microdomains. P. Spearman's lab (Dong et al.) reported interesting findings implicating the AP-3 clathrin adaptor protein complex (which among other things regulates CD63 trafficking) in HIV-1 Gag targeting. They observed that the δ subunit of AP-3 interacts with the MA domain of Gag and that either overexpression of an N-terminal δ subunit fragment or siRNA knockdown of AP-3 δ subunit expression inhibits HIV-1 release.

H. Wang from L. Mansky's group observed that HTLV-1 assembly, like that of HIV-1, can take place in the MVB. Interestingly, while in HeLa cells HTLV-1 assembly appears to occur primarily at the plasma membrane, mutational disruption of the Pro-Thr-Ala-Pro (PTAP) late domain results in accumulation of virus particles in the MVB, consistent with published results (e.g., [24]). T. Hope's lab (Gomez and Hope) confirmed the finding [25] that HIV-1 mutants lacking the p6 late domain still assemble in MVBs in macrophages, indicating that interactions between p6 and cellular host factors (e.g., Tsg101) are not required for MVB localization. Reports from the labs of D. Muriaux (Grigorov et al.) and F.-L. Cosset (Sandrin et al.) suggested that interactions between MLV Gag and Env may occur in an intracellular compartment and that this interaction might influence both Gag trafficking and Env incorporation into virions. Some of this work has now been published [26].

A study from the Freed lab (Ono and Freed) examined the possibility that the phosphoinositide phosphatidylinositol-(4,5)-bisphosphate (PI(4,5)P_2_) plays a role in Gag targeting. This lipid is of interest since it is involved in the trafficking of a variety of cellular proteins that, like many retroviral Gag proteins, contain a basic membrane binding domain. Furthermore, work from A. Rein's lab [27] has shown that, *in vitro*, HIV-1 Gag binds molecules structurally related to phosphoinositides. A. Ono used enzymatic approaches to perturb cellular PI(4,5)P_2 _levels in virus-expressing cells and observed that Gag targeting and virus assembly were shifted from the plasma membrane to intracellular compartments. These results indicate that cellular PI(4,5)P_2 _plays a role in directing Gag to the plasma membrane.

Work from J. Lingappa's lab previously suggested the involvement of the cellular RNase L inhibitor HP68 in HIV-1 assembly [28]. This work was extended at the meeting by Dooher et al., who showed that HP68, Gag, and genomic RNA colocalize in virus-producing cells. The cellular protein nucleolin was also found to be a component of the Gag/HP68 complex.

Novel findings relating to the regulation of foamy virus (FV) release were reported by the Lindemann lab. FVs are unusual among retroviruses in that Env glycoprotein expression plays a critical role in particle release. The leader peptide of FV Env, which is generated by a cleavage event during Env trafficking to the plasma membrane and is incorporated into particles, seems to play an important role in the budding process. Lindemann et al. reported the identification of ubiquitylated forms of the leader peptide; suppression of these ubiquitylated forms markedly stimulated subviral particle release. These results suggest that ubiquitylation of the FV Env leader peptide modulates the ratio of particle vs. subviral particle budding.

Progress continues to be made in visualizing retrovirus assembly and release in real time in living cells. In addition to use of the tetracyteine/biarsenical live-cell labeling system described above, B. Muller from H.-G. Krausslich's lab discussed the development of an infectious HIV-1 derivative containing a GFP insert near the C-terminus of MA. This derivative produces particles with wild-type morphology; however, release kinetics are impaired. While the Gag-GFP virus is poorly infectious, infectivity can be restored upon coexpression with wild-type HIV-1. This Gag-GFP HIV-1 derivative should be useful for both assembly/release and post-entry studies. In a presentation from the labs of V. Vogt and W. Webb, D. Larson described the use of correlated fluorescence microscopy and scanning electron microscopy to visualize Rous sarcoma virus (RSV) budding in real time using a GagGFP derivative.

Major advances have been made in the past three years in elucidating the host cell machinery required for the release of retrovirus particles from infected cells [29,30]. It is now well accepted that retroviruses use their late domains to commandeer machinery that normally plays a central role in promoting the budding of vesicles into the MVB. This machinery includes the so-called "class E Vps" factors originally identified in yeast as being crucial for MVB biogenesis [31]. Many of these class E Vps proteins are found in three multisubunit complexes known as ESCRT-I, -II, and -III. Retroviral late domains come in three flavors: Pro-Thr/Ser-Ala-Pro [P(T/S)AP], Pro-Pro-x-Tyr (PPxY), and Tyr-Pro-Asp-Leu (YPDL). HIV-1 release is controlled predominantly by a P(T/S)AP-type late domain; many retroviruses, including MLV and RSV, contain PPxY late domains; and equine infectious anemia virus (EIAV) harbors a YPDL late domain. Several retroviruses [e.g., Mason-Pfizer monkey virus (M-PMV) and HTLV-1] encode both P(T/S)AP and PPxY late domains. In addition to its P(T/S)AP motif, HIV-1 contains a secondary YPDL-related sequence whose role in HIV-1 release remains to be defined. It is now well established that the P(T/S)AP motif interacts with the ESCRT-I component Tsg101. Recent data from several labs (those of H. Gottlinger, W. Sundquist, and P. Bieniasz) have strongly suggested that AIP1 (also known as ALIX) is the host factor with which YPDL interacts. There is less certainty about the identity of the biologically relevant PPxY-interacting protein. PPxY motifs in cellular proteins often interact with WW domains, and several PPxY-containing retroviral Gag proteins have been reported to bind the ubiquitin ligase Nedd4 (or related proteins), which contains a series of centrally located WW domains. P. Bieniasz's lab (Martin-Serrano et al.) reported at the meeting that MLV Gag binds the Nedd4-related proteins WWP1 (which has also been shown to associate with HTLV-1 Gag [32]), WWP2 and ITCHY. The extent to which binding to these proteins was reduced by mutations in the PPPY motif correlated with the severity of the budding defect induced by the mutations. The authors also observed that WWP1 localizes to aberrant endosomes induced by expression of a dominant-negative Vps4 in a manner dependent on the ubiquitin ligase (or HECT) domain, suggesting that the HECT domain may link WWP1 (and consequently Gag) to the class E Vps machinery. J. Leis's lab previously reported that overexpression of the WW domain-containing region from a chicken Nedd4-like protein inhibited RSV particle production. At this year's meeting, this work was extended (Vana et al.) by showing that in cells overexpressing this WW domain-containing fragment RSV virions accumulated in intracellular inclusion bodies. Interestingly, V. Vogt's lab (Johnson et al.) found that overexpression of the C-terminal domain of Tsg101 formed aggresome-like structures that trapped HIV-1 Gag.

In yeast, ESCRT-I contains three protein components: Vps23 (the yeast homolog of Tsg101), Vps28 and Vps37. In mammalian cells, only the Vps23 and Vps28 homologs had been identified. At this year's meeting, M. Stuchell from W. Sundquist's lab reported the cloning of human Vps37. As in yeast, human Vps37 binds Tsg101 and is present along with Tsg101 and Vps28 in a high molecular weight ESCRT-I complex. Much of this work has recently been published [33], as have similar results from H. Stenmark's lab [34]. Further illustrating the importance of ESCRT-I in HIV-1 budding, fusion of Vps37 to the C-terminus of Gag reverses the release defect imposed by PTAP deletion [33]. Y. Yardin's lab (Amit et al.) described the identification of an E3 ubiquitin ligase (termed Tal, for Tsg101-associated ligase) that binds the N-terminal UEV domain of Tsg101 and apparently regulates its activity. This work has also recently been published [35].

M. Palmarini's lab (Mura et al.) described a novel type of retroviral interference that operates at the level of virus assembly and release. The sheep genome harbors a number of copies of endogenous retroviruses closely related to the pathogenic exogenous β-retrovirus Jaagsiekte sheep retrovirus (JSRV). One of these endogenous retroviruses (enJS56A1) displays a defect in assembly/release. EM analysis indicates that enJS56A1 particles form large perinuclear aggregates; these appear to trap JSRV particles intracellularly when enJS56A1 and JSRV are coexpressed. The authors speculate that enJS56A1 may have helped protect sheep from infection with related, exogenous retroviruses during evolution. Much of this study has recently been published [36].

It has been known for many years that several retroviruses express two forms of Gag: a conventional version and a larger, glycosylated form referred to as glycoGag. GlycoGag is synthesized using an alternative, upstream initiation codon, and unlike its smaller counterpart it is transported to the plasma membrane through the secretory pathway. H. Fan's lab (Low et al.) reported at this year's meeting that a packaging cell line that expresses Gag without glycoGag produces tube-shaped particles at the cell surface. This apparent assembly defect was corrected by coexpression of glycoGag. The expression of glycoGag increased both virus yield and infectivity. These results suggest that glycoGag may function, in a manner that remains to be elucidated, in regulating proper virus assembly and release.

## Integration

Retroviral preintegration complexes (PICs) and the integrase (IN) enzyme itself, interact with a variety of host factors during nuclear import of the PIC and integration of the newly synthesized viral DNA into the host cell chromosome. One such host factor, lens epithelium-derived growth factor (LEDGF/p75), was recently reported to bind HIV-1 IN [37,38]. Several presentations at this year's meeting provided varied and conflicting results regarding the role of LEDGF/p75 in the integration process. S. Emiliani reported data from a collaboration between the labs of R. Benarous and Z. Debyser showing that knockdown of LEDGF/p75 expression using siRNA in HeLa P4 cells inhibited HIV-1 replication without affecting the import of IN to the nucleus. The Q168L mutation in HIV-1 IN abolished both virus replication and the interaction between IN and LEDGF/p75. Busschots from the Debyser lab showed that the interaction of LEDGF/p75 with IN increased the affinity of IN for DNA. Based on these data, it was postulated that LEDGF/p75 may play a role in tethering IN to chromosomal DNA. E. Poeschla's lab (Llano et al., also see [39]) observed that endogenous LEDGF/p75 co-immunoprecipitated with both HIV-1 and FIV IN. Stable knock-down of LEDGF/p75 expression in 293T cells induced a redistribution of both HIV-1 and FIV IN from the nucleus to the cytoplasm but apparently did not affect nuclear import of HIV-1 or FIV PICs since lentiviral vector infectivity was not reduced under these conditions. Furthermore, stable knock-down of LEDGF/p75 expression in the Jurkat T-cell line did not affect HIV-1 replication. However, LEDGF/p75 was found to be a component of lentiviral PICs. The authors concluded that LEDGF/p75 is not required for lentiviral integration but advanced the hypothesis that it might play a role in target site selection. Vandekerckhove and colleagues from the Debyser lab reported that stable knock-down of LEDGF/p75 expression in HeLa P4 or MOLT cells delayed HIV-1 replication but did not diminish infectivity mediated by a VSV-G pseudotyped lentiviral vector. The authors controlled for non-specific siRNA effects by using double mismatched siRNA. Results from A. Engleman and coworkers (Vandegraaff et al.) raised further questions concerning the role of LEDGF/p75 in HIV-1 integration. They observed that while two LEDGF/p75 siRNAs reduced LEDGF/p75 protein levels, only the one originally described by the Debyser lab impaired HIV-1 infectivity. This infectivity defect could not be reversed by partially restoring LEDGF/p75 expression. Based on these results, the authors cautioned that the effect of LEDGF/p75 siRNA on HIV-1 infectivity may be due to non-specific effects not directly related to LEDGF/p75. Clearly, additional studies need to be performed to clarify the role of LEDGF/p75 in lentiviral integration.

A number of presentations focused on the target site specificity of retroviral (or retrotransposon) integration. Different retroviruses and retroelements display strong preferences in selecting their target sites in the genomes of their host cells (for review see [40]). For example, the Ty1 and Ty3 retrotransposons integrate predominantly upstream of Pol III-transcribed genes; the Tf1 retrotransposon selects sequences upstream of Pol II-transcribed genes; HIV-1 tends to integrate in actively transcribed regions; and MLV prefers to integrate in the promoters of active genes. At this year's meeting, A. Narezkina from R. Katz's and A. Skalka's lab, and R. Mitchell from F. Bushman's group, both described the results of genome-wide analyses of ASLV integration sites. They observed that, unlike MLV, this avian retrovirus does not prefer to integrate at transcription start sites, and unlike HIV-1 does not display a strong preference for highly active genes. Both groups did observe a relatively modest tendency for integration within genes. H. Levin's group (Kelly et al.) extended their previous work on Tf1 integration. Using a target plasmid containing a single gene, they observed that nearly all integration events took place in the gene's promoter region. Interestingly, the integration sites displayed a periodic pattern in which integrated copies of Tf1 were separated by around 30 nucleotides. This targeting appeared to be dependent on promoter activity. Kelly and coworkers speculated that the chromodomain in the Tf1 IN binds histones and regulates integration into specific targets in a promoter-positioned nucleosome. Holman and Coffin reported on the analysis of base preferences immediately surrounding integrated HIV-1 proviruses. Using a large amount of data derived from previously reported integration sites, the authors observed strong base preferences within seven residues at either end of integrated proviruses. The presentation emphasized that HIV-1 integration shows target preferences on both a macro scale (as noted above) and on a microscale, involving the residues immediately adjacent to the integration site.

M. Katzman's lab (Konsavage et al.) reported an interesting RSV IN mutant that displayed enhanced 3'-end processing activity but impaired DNA joining. The authors speculated that RSV IN has evolved suboptimal processing activity to allow it to catalyze DNA joining.

R. Craigie's lab previously reported that a protein termed "barrier-to-autointegration factor" (BAF) is a component of the MLV preintegration complex and that this factor enhances intermolecular integration reactions and blocks intramolecular integration (autointegration) [41]. At this year's meeting, Suzuki and Craigie extended this work by showing that BAF interacts with an inner nuclear membrane protein, lamina-associated polypeptide 2α (LAP2α). LAP2α is a component of the MLV preintegration complex and knockdown of its expression inhibits MLV replication.

## Reverse Transcription

A number of studies over the years have demonstrated that retroviral NC proteins possess nucleic acid chaperone activity that plays an important role in various aspects of reverse transcription (for review, see [42]). Several presentations focused on this activity of NC. Work from J. Levin's lab (Guo et al.) demonstrated that in an *in vitro *assay that models minus-strand transfer, NC alone is able to catalyze the removal of small 5' terminal genomic RNA fragments, which remain annealed to a minus-strand strong-stop DNA. Strand transfer product increased with increasing NC, and in the presence of NC, strand transfer product was generated even when reverse transcription was catalyzed by an RNase H-deficient RT. The NC zinc fingers appeared to be critical for this activity. These results suggest an important role for NC nucleic acid chaperone activity in removing terminal RNA fragments annealed to minus-strand strong-stop DNA following primary cleavage by RNase H. By examining reverse transcription in infected cells, R. Gorelick's lab (Thomas et al.) observed that mutations in the first zinc finger of NC strongly interfered with the progression of reverse transcription and impaired virus infectivity. The authors speculated that reduced binding of NC to the viral DNA allows cellular enzymes (nucleases and ligases) to modify the viral DNA ends thereby interfering with integration.

Reverse transcription is initiated by a host cell-derived tRNA bound to the primer binding site (PBS) near the 5' end of the viral genome. Retroviruses are selective in their utilization of host tRNAs; HIV-1, for example, specifically primes reverse transcription with a tRNA^lys3^. The selectivity for particular tRNAs results from interactions between the tRNA and the PBS and it has been proposed by B. Berkhout and colleagues that a second motif in the viral RNA, termed the primer-activation signal (PAS), also forms specific contacts with the tRNA. Berkhout's lab (Abbink et al.) reported at this year's meeting that, by mutagenesis of the PBS and PAS, HIV-1 primer utilization could be shifted from tRNA^lys3 ^to tRNA^lys1,2^. Such mutants replicated poorly but could adapt during long-term passaging. In one case, adaptation evidently occurred through optimization of the putative PAS. Interestingly, a single amino acid change in the RNase H domain of RT also arose during adaptation, suggesting a possible role for RT in selective primer utilization.

While PCR techniques allow the progression of viral DNA synthesis to be monitored post-infection, it has not been possible to follow reverse transcription in a strand-specific fashion in infected cells. D. Thomas from V. Pathak's lab reported the development of a strand-specific amplification assay that uses so-called "padlock" probes – long single-stranded oligonucleotides that hybridize with their target sequence simultaneously at their 5' and 3' ends. Following hybridization, the ends of the padlock probes are ligated to form circles that are amplified and detected by real-time PCR. This assay, which allows specific steps in reverse transcription to be measured quantitatively in a strand-specific manner, should be very useful for addressing a number of questions regarding the kinetics of reverse transcription and the efficiency of this process under a variety of conditions.

F. Maldarelli described the results of studies conducted with S. Palmer, J. Coffin and colleagues aimed at characterizing the evolution of HIV-1 populations *in vivo*. The authors monitored genetic variation in cohorts of drug-naïve and drug-resistant patients by analyzing individual *pro-pol *sequences. In both drug-naïve and drug-resistant patients, they observed little change in virus population structure over several years, implying that the replicating population is relatively large. Interestingly, recombination rates appeared to be very high regardless of levels of viremia, suggesting the presence of a substantial number of multiply infected cells even at low viral loads. The results emphasize the importance of recombination in generating viral diversity *in vivo*.

## Pathogenesis

The keynote speaker at this year's meeting was Neal Copeland, who presented important findings from his and Nancy Jenkins' lab regarding the use of high through-put analysis to map retroviral integration sites in tumors induced by MLVs. This rapidly developing approach is being used by a number of groups to elucidate cancer gene pathways [43-46], and to define retroviral integration site preferences. The Jenkins and Copeland labs have used infection of various hematopoietic stem cells followed by transplantation into lethally irradiated mice to identify not only novel proto-oncogenes, but also cooperating cancer genes, tumor suppressors, and genes involved in stem cell transformation and immortalization. While this technique is currently applicable only to hematopoietic lineage cells, current research in the Jenkins and Copeland labs is testing whether integration by transposable elements in other cell types can be used to identify similar genes and pathways in solid tumors.

In contrast to the majority of leukemia-inducing retroviruses in non-human species, most of which cause cancer by insertional mutagenesis, HTLV-I encodes accessory proteins, such as Tax, with known transforming activity. There were several talks at the meeting describing new accessory proteins that may also play a role in cell regulation. Two talks focused on the protein p12^I^, which is targeted to the endoplasmic reticulum/Golgi of infected cells and decreases MHC class I trafficking to the cell surface. R. Fukomoto from G. Franchini's lab presented data that p12^I ^decreases activation of the transcription factor NFATI in T cells by binding to LAT and inhibiting T-cell receptor signaling. In contrast, M. Lairmore presented data that p12^I ^expression in Jurkat cells results in ~20-fold activation of NFAT-dependent gene expression in a calcium-dependent manner (Kim and Lairmore). These authors also demonstrated that p12^I ^acts in the endoplasmic reticulum to activate calcium-mediated T-cell activation during the early stages of infection, apparently through an interaction with calcineurin. These studies suggest a prominent role for p12^I ^in common T cell activation pathways critical to the establishment of a persistent infection. Another protein, p13^II^, which was described by V. Ciminale (Silic-Benussi et al.), is localized to the inner mitochondrial membrane and induces changes in Ca^2+ ^and K^+ ^permeability. In culture, p13^II ^reverses the morphological transformation of rat embryo fibroblasts expressing c-Myc and Ha-Ras and decreases their ability to form tumors in nude mice. There was speculation that HTLV-I encodes p13^II ^to counteract the growth-inducing properties of the other viral accessory proteins (such as Tax) that are required for establishment of infection, thereby allowing the virus to persist in infected individuals.

In a theme that echoed in several of the simple retrovirus talks, V. Armbruester from the Mueller-Lantzsch laboratory reported on a novel protein generated by alternative splicing of the envelope gene of the HERV-K endogenous retrovirus. The transcript for this protein, np9, is highly expressed in mammary carcinomas and germ cells, and the gene product binds to the LNX protein, which is a ligand of Numb and targets it for proteasomal degradation. Since LNX/Numb/Notch is a known transformation pathway in tumors, Armbruester speculated that np9 may play a role in tumorigenesis by sequestering LNX, thereby stabilizing Numb.

## RNA transcription, processing, export and packaging

This session surveyed new findings on retroviral splicing, nuclear retention and export, translation and packaging. J. Madsen and M. Stoltzfus evaluated the role of an exonic splicing silencer (ESS) on HIV-1 replication in cultured T cells. An HIV-1 mutant with ESS substitutions displays a replication-defective phenotype that correlates with increased viral RNA splicing. This mutant was subjected to long-term passage and the viruses that emerged contained second-site reversions in splice sites flanking the exon containing the mutated ESS. Stoltzfus speculated that strains that do not contain the ESS maintain balanced expression of their viral genome by a novel, unknown mechanism.

A. Lever's lab (Poole et al.) described confocal microscopy and fluorescence resonance energy transfer (FRET) analysis of the interaction between HIV-1 Gag and its cognate genomic RNA. Using biotinylated probes to the full-length, unspliced RNA, he described an initial perinuclear co-localization between Gag and genomic RNA that subsequently shifted to the plasma membrane. Mutation of the Ψ packaging signal partially disrupted the perinuclear and plasma membrane colocalization.

J. Dudley's lab (J. Mertz et al.) described a new MMTV gene that encodes a viral RNA transport protein generated by alternative splicing of the *env *gene. The gene was designated *rem*, (for RNA export protein of MMTV RNA). Its product stimulated nucleocytoplasmic transport of the unspliced MMTV transcript, in a manner similar that of the Rec protein of HERV-K [47,48]. This finding indicates that MMTV encodes at least three accessory genes (encoding dUTPase, superantigen, and Rem) in addition to the standard retroviral genes (*gag*, *pol*, and *env*). The Dudley group suggested that since MMTV exhibits a complex genetic structure it should be reclassified as a complex retrovirus.

K. Boris-Lawrie (Roberts et al.) presented genetic and biochemical data showing that the R-U5 region of SNV RNA adopts a stem-loop structure that stimulates cap-dependent translation. RNA affinity and proteomic analysis showed that RNA helicase A (RHA) bound to wild-type RNA but not to mutants containing substitutions in this structure. RHA interaction with SNV R-U5 stimulated translation of unspliced HIV-1 reporter mRNA. Boris-Lawrie speculated that this could occur by rearrangement of intramolecular RNA interactions that disrupt the packaging signal, thus facilitating mRNA translation.

*In vivo*, only a fraction of HTLV-infected cells actively expresses viral RNA, leading to speculation that the virus negatively regulates gene expression. P. Green (Younis et al.) described a new role for HTLV-1 and -II accessory proteins p30 and p28, respectively, in negatively regulating virus production during chronic infection. Tax/Rex expression was inhibited upon ectopic expression of p28 by a mechanism that involved nuclear retention of the mRNA. Analysis of RNAs bearing a luciferase reporter gene showed that the 3' splice junction was sufficient to confer this nuclear retention. From this work and the studies from the Franchini and Lairmore labs described above, it is clear that HTLV-1 has evolved to regulate its expression in infected cells, thereby evading immune recognition and promoting viral persistence.

## Antivirals

As in previous years, there was a strong emphasis on the development of antiviral agents that interfere with reverse transcription. However, noteworthy progress was also reported in efforts to target a number of additional steps in the replication cycle.

S. Sarafianos reported data from E. Arnold's lab (Himmel et al.) on the structure of HIV-1 RT in a complex with an RNase H inhibitor. Interestingly, the binding site of the compound is quite distant (>40 Angstroms) from the RNase H active site and partially overlaps the NNRTI binding pocket. These results raise the intriguing possibility that compounds could be designed that simultaneously act as RNase H inhibitors and as NNRTIs. M. Miller's lab (Shaw-Reid et al.) performed *in vitro *assays to examine the effect of RT polymerase inhibitors on RNase H activity. They observed that NNRTIs actually increased RNase H activity; structural studies suggested that this enhancement was due to greater accessibility of the DNA/RNA duplex by RNase H. Although a diketo acid RNase H inhibitor displayed decreased potency in the presence of an NNRTI, the diketo acid and NNRTI synergistically inhibited reverse transcription overall.

C. Wild, E. Freed, and colleagues, and independently C. Aiken's lab, previously reported that a dimethyl succinyl betulinic acid derivative (referred to as PA-457 or DSB) potently blocks HIV-1 infectivity by specifically disrupting the cleavage of the CA precursor (composed of CA and spacer peptide SP1) to mature CA [49,50]. The block to CA-SP1 processing prevents proper core condensation in virions released from PA-457-treated cells. Interestingly, HIV-2 and SIV are insensitive to PA-457. This work was extended by the labs of C. Wild and E. Freed (F. Li et al.) to demonstrate that the determinant of PA-457 activity maps to the N-terminus of SP1. In addition, (Adamson et al.) the passaging of HIV-1 at sub-optimal concentrations of PA-457 led to the appearance of PA-457-resistant variants that contain mutations in the C-terminus of CA or the N-terminus of SP1.

A. Lever's lab (Brown et al.) described their efforts to inhibit HIV-1 replication using oligonucleotides that target the viral genome. The authors observed that oligos targeting the packaging signal (specifically stem-loops 2 and 3) disrupt Gag binding and reduced virus infectivity. T. Murakami and coworkers reported the development of an orally bioavailable compound that binds the HIV-1 chemokine coreceptor CXCR4. In culture, the compound potently inhibits infection by HIV-1 isolates that use CXCR4 as a coreceptor, but, as expected, do not block infection by strains that exclusively use CCR5 as a coreceptor. The compound suppressed HIV-1 infection in the hu-PBL-SCID mouse model. The results of this study suggest that this CXCR4 antagonist could potentially be an effective drug in infected humans.

It has been suggested that most virus originating in the central nervous system (CNS) derives from long-lived cells (e.g., macrophages) that would continue to produce virus for a significant period of time after the initiation of antiretroviral therapy. According to this model, CNS-derived virus should decay more slowly following the onset of therapy relative to virus derived from the blood. In the last presentation of the conference, data were presented from R. Swanstrom's lab (Harrington et al.) obtained from a study of HIV-1 population dynamics in cerebrospinal fluid (CSF) immediately following the initiation of antiretroviral therapy. Virus isolates in the CSF apparently derive from both the CNS and the blood plasma. Interestingly, using heteroduplex tracking assays, the authors observed that within the first several days following the initiation of therapy CNS-derived isolates in the CSF decline with similar kinetics to isolates shared with the blood, suggesting that virus from both compartments is produced by cells with a short life span.

## References

[B1] Manel N, Kim FJ, Kinet S, Taylor N, Sitbon M, Battini JL (2003). The ubiquitous glucose transporter GLUT-1 is a receptor for HTLV. Cell.

[B2] Manel N, Taylor N, Kinet S, Kim FJ, Swainson L, Lavanya M, Battini JL, Sitbon M (2004). HTLV envelopes and their receptor GLUT-1, the ubiquitous glucose transporter: a new vision on HTLV infection?. Front Biosci.

[B3] Shimojima M, Miyazawa T, Ikeda Y, McMonagle EL, Haining H, Akashi H, Takeuchi Y, Hosie MJ, Willett BJ (2004). Use of CD134 as a primary receptor by the feline immunodeficiency virus. Science.

[B4] Lavillette D, Ruggieri A, Russell SJ, Cosset FL (2000). Activation of a cell entry pathway common to type C mammalian retroviruses by soluble envelope fragments. J Virol.

[B5] Barnett AL, Cunningham JM (2001). Receptor binding transforms the surface subunit of the mammalian C-type retrovirus envelope protein from an inhibitor to an activator of fusion. J Virol.

[B6] Katane M, Fujita R, Takao E, Kubo Y, Aoki Y, Amanuma H (2004). An essential role for the His-8 residue of the SDF-1alpha-chimeric, tropism-redirected Env protein of the Moloney murine leukemia virus in regulating postbinding fusion events. J Gene Med.

[B7] Wu BW, Lu J, Gallaher TK, Anderson WF, Cannon PM (2000). Identification of regions in the Moloney murine leukemia virus SU protein that tolerate the insertion of an integrin-binding peptide. Virology.

[B8] Wallin M, Ekstrom M, Garoff H (2004). Isomerization of the intersubunit disulphide-bond in Env controls retrovirus fusion. Embo J.

[B9] Aguilar HC, Anderson WF, Cannon PM (2003). Cytoplasmic tail of Moloney murine leukemia virus envelope protein influences the conformation of the extracellular domain: implications for mechanism of action of the R Peptide. J Virol.

[B10] Rothenberg SM, Olsen MN, Laurent LC, Crowley RA, Brown PO (2001). Comprehensive mutational analysis of the Moloney murine leukemia virus envelope protein. J Virol.

[B11] Geijtenbeek TB, Kwon DS, Torensma R, van Vliet SJ, van Duijnhoven GC, Middel J, Cornelissen IL, Nottet HS, KewalRamani VN, Littman DR, Figdor CG, van Kooyk Y (2000). DC-SIGN, a dendritic cell-specific HIV-1-binding protein that enhances trans-infection of T cells. Cell.

[B12] Sheehy AM, Gaddis NC, Choi JD, Malim MH (2002). Isolation of a human gene that inhibits HIV-1 infection and is suppressed by the viral Vif protein. Nature.

[B13] Schrofelbauer B, Chen D, Landau NR (2004). A single amino acid of APOBEC3G controls its species-specific interaction with virion infectivity factor (Vif). Proc Natl Acad Sci U S A.

[B14] Xu H, Svarovskaia ES, Barr R, Zhang Y, Khan MA, Strebel K, Pathak VK (2004). A single amino acid substitution in human APOBEC3G antiretroviral enzyme confers resistance to HIV-1 virion infectivity factor-induced depletion. Proc Natl Acad Sci U S A.

[B15] Bieniasz PD (2003). Restriction factors: a defense against retroviral infection. Trends Microbiol.

[B16] Lilly F (1967). Susceptibility to two strains of Friend leukemia virus in mice. Science.

[B17] Stremlau M, Owens CM, Perron MJ, Kiessling M, Autissier P, Sodroski J (2004). The cytoplasmic body component TRIM5alpha restricts HIV-1 infection in Old World monkeys. Nature.

[B18] Hatziioannou T, Perez-Caballero D, Yang A, Cowan S, Bieniasz PD (2004). Retrovirus resistance factors Ref1 and Lv1 are species-specific variants of TRIM5alpha. Proc Natl Acad Sci U S A.

[B19] Best S, Le Tissier P, Towers G, Stoye JP (1996). Positional cloning of the mouse retrovirus restriction gene Fv1. Nature.

[B20] Sayah DM, Sokolskaja E, Berthoux L, Luban J (2004). Cyclophilin A retrotransposition into TRIM5 explains owl monkey resistance to HIV-1. Nature.

[B21] Narayan S, Young JA (2004). Reconstitution of retroviral fusion and uncoating in a cell-free system. Proc Natl Acad Sci U S A.

[B22] Gaietta G, Deerinck TJ, Adams SR, Bouwer J, Tour O, Laird DW, Sosinsky GE, Tsien RY, Ellisman MH (2002). Multicolor and electron microscopic imaging of connexin trafficking. Science.

[B23] Nydegger S, Foti M, Derdowski A, Spearman P, Thali M (2003). HIV-1 egress is gated through late endosomal membranes. Traffic.

[B24] Bouamr F, Melillo JA, Wang MQ, Nagashima K, de Los Santos M, Rein A, Goff SP (2003). PPPYVEPTAP motif is the late domain of human T-cell leukemia virus type 1 Gag and mediates its functional interaction with cellular proteins Nedd4 and Tsg101 [corrected]. J Virol.

[B25] Ono A, Freed EO (2004). Cell-type-dependent targeting of human immunodeficiency virus type 1 assembly to the plasma membrane and the multivesicular body. J Virol.

[B26] Sandrin V, Muriaux D, Darlix JL, Cosset FL (2004). Intracellular trafficking of Gag and Env proteins and their interactions modulate pseudotyping of retroviruses. J Virol.

[B27] Campbell S, Fisher RJ, Towler EM, Fox S, Issaq HJ, Wolfe T, Phillips LR, Rein A (2001). Modulation of HIV-like particle assembly in vitro by inositol phosphates. Proc Natl Acad Sci U S A.

[B28] Zimmerman C, Klein KC, Kiser PK, Singh AR, Firestein BL, Riba SC, Lingappa JR (2002). Identification of a host protein essential for assembly of immature HIV-1 capsids. Nature.

[B29] Pornillos O, Garrus JE, Sundquist WI (2002). Mechanisms of enveloped RNA virus budding. Trends Cell Biol.

[B30] Freed EO (2002). Viral late domains. J Virol.

[B31] Katzmann DJ, Odorizzi G, Emr SD (2002). Receptor downregulation and multivesicular-body sorting. Nat Rev Mol Cell Biol.

[B32] Heidecker G, Lloyd PA, Fox K, Nagashima K, Derse D (2004). Late assembly motifs of human T-cell leukemia virus type 1 and their relative roles in particle release. J Virol.

[B33] Stuchell MD, Garrus JE, Muller B, Stray KM, Ghaffarian S, McKinnon R, Krausslich HG, Morham SG, Sundquist WI (2004). The human endosomal sorting complex required for transport (ESCRT-I) and its role in HIV-1 budding. J Biol Chem.

[B34] Bache KG, Slagsvold T, Cabezas A, Rosendal KR, Raiborg C, Stenmark H (2004). The Growth-Regulatory Protein HCRP1/hVps37A Is a Subunit of Mammalian ESCRT-I and Mediates Receptor Down-Regulation. Mol Biol Cell.

[B35] Amit I, Yakir L, Katz M, Zwang Y, Marmor MD, Citri A, Shtiegman K, Alroy I, Tuvia S, Reiss Y, Roubini E, Cohen M, Wides R, Bacharach E, Schubert U, Yarden Y (2004). Tal, a Tsg101-specific E3 ubiquitin ligase, regulates receptor endocytosis and retrovirus budding. Genes Dev.

[B36] Mura M, Murcia P, Caporale M, Spencer TE, Nagashima K, Rein A, Palmarini M (2004). Late viral interference induced by transdominant Gag of an endogenous retrovirus. Proc Natl Acad Sci U S A.

[B37] Maertens G, Cherepanov P, Pluymers W, Busschots K, De Clercq E, Debyser Z, Engelborghs Y (2003). LEDGF/p75 is essential for nuclear and chromosomal targeting of HIV-1 integrase in human cells. J Biol Chem.

[B38] Cherepanov P, Maertens G, Proost P, Devreese B, Van Beeumen J, Engelborghs Y, De Clercq E, Debyser Z (2003). HIV-1 integrase forms stable tetramers and associates with LEDGF/p75 protein in human cells. J Biol Chem.

[B39] Llano M, Vanegas M, Fregoso O, Saenz D, Chung S, Peretz M, Poeschla EM (2004). LEDGF/p75 determines cellular trafficking of diverse lentiviral but not murine oncoretroviral integrase proteins and is a component of functional lentiviral preintegration complexes. J Virol.

[B40] Bushman FD (2003). Targeting survival: integration site selection by retroviruses and LTR-retrotransposons. Cell.

[B41] Lee MS, Craigie R (1994). Protection of retroviral DNA from autointegration: involvement of a cellular factor. Proc Natl Acad Sci U S A.

[B42] Rein A, Henderson LE, Levin JG (1998). Nucleic-acid-chaperone activity of retroviral nucleocapsid proteins: significance for viral replication. Trends Biochem Sci.

[B43] Lund AH, Turner G, Trubetskoy A, Verhoeven E, Wientjens E, Hulsman D, Russell R, DePinho RA, Lenz J, van Lohuizen M (2002). Genome-wide retroviral insertional tagging of genes involved in cancer in Cdkn2a-deficient mice. Nat Genet.

[B44] Mikkers H, Allen J, Knipscheer P, Romeijn L, Hart A, Vink E, Berns A, Romeyn L (2002). High-throughput retroviral tagging to identify components of specific signaling pathways in cancer. Nat Genet.

[B45] Suzuki T, Shen H, Akagi K, Morse HC, Malley JD, Naiman DQ, Jenkins NA, Copeland NG (2002). New genes involved in cancer identified by retroviral tagging. Nat Genet.

[B46] Kim R, Trubetskoy A, Suzuki T, Jenkins NA, Copeland NG, Lenz J (2003). Genome-based identification of cancer genes by proviral tagging in mouse retrovirus-induced T-cell lymphomas. J Virol.

[B47] Magin C, Hesse J, Lower J, Lower R (2000). Corf, the Rev/Rex homologue of HTDV/HERV-K, encodes an arginine-rich nuclear localization signal that exerts a trans-dominant phenotype when mutated. Virology.

[B48] Magin-Lachmann C, Hahn S, Strobel H, Held U, Lower J, Lower R (2001). Rec (formerly Corf) function requires interaction with a complex, folded RNA structure within its responsive element rather than binding to a discrete specific binding site. J Virol.

[B49] Li F, Goila-Gaur R, Salzwedel K, Kilgore NR, Reddick M, Matallana C, Castillo A, Zoumplis D, Martin DE, Orenstein JM, Allaway GP, Freed EO, Wild CT (2003). PA-457: a potent HIV inhibitor that disrupts core condensation by targeting a late step in Gag processing. Proc Natl Acad Sci U S A.

[B50] Zhou J, Yuan X, Dismuke D, Forshey BM, Lundquist C, Lee KH, Aiken C, Chen CH (2004). Small-molecule inhibition of human immunodeficiency virus type 1 replication by specific targeting of the final step of virion maturation. J Virol.

